# Dietary Fermented Pineapple Pomace Improves Intestinal Health and Antioxidant Capacity Without Compromising Growth Performance in Juvenile Largemouth Bass (*Micropterus salmoides*)

**DOI:** 10.3390/ani16152316

**Published:** 2026-07-28

**Authors:** Cong Wang, Like Zhang, Chunfeng Yao, Beiping Tan, Shiwei Xie, Shuyan Chi, Hongyu Liu, Junming Deng

**Affiliations:** 1 College of Fisheries, Guangdong Ocean University, Zhanjiang 524088, China; wc9264@163.com (C.W.); likeeee2020@163.com (L.Z.); bptan@126.com (B.T.); xswzsdx@163.com (S.X.); chishuyan77@163.com (S.C.); liu_hong_yu@163.com (H.L.); 2 Guangdong Yuehai Feeds Group Co., Ltd., Zhanjiang 524017, China; xiaoyao3221@163.com

**Keywords:** largemouth bass, fermented pineapple pomace, intestinal health, antioxidant capacity

## Abstract

This study evaluated the influence of dietary fermented pineapple pomace (FPP) supplementation on growth performance, intestinal health, and antioxidant capacity in juvenile largemouth bass (*Micropterus salmoides*). Six experimental diets containing 0%, 2%, 4%, 6%, 8%, and 10% FPP were formulated and fed for 10 weeks, with FPP replacing equivalent proportions of wheat flour in the basal diet. The results showed that dietary FPP did not impair the growth performance of largemouth bass. The FP4 and FP6 diets significantly increased the villus height, whereas the FP4, FP6, and FP8 diets significantly increased the villus width. Dietary FPP also enhanced several intestinal digestive and absorptive enzyme activities, including lipase, amylase, trypsin, lactase, and alkaline phosphatase, although the responsive inclusion levels differed among enzymes. Dietary FPP generally reduced intestinal mucosal permeability, alleviated barrier damage, and enhanced mucosal barrier integrity. In addition, FPP supplementation improved intestinal chemical and immune barrier function, as reflected by increased lysozyme activity, mucin 2 level, secretory immunoglobulin T level, and immunoglobulin M level at appropriate inclusion levels. Dietary FPP improved antioxidant status by increasing serum catalase activity and total antioxidant capacity at appropriate inclusion levels, while low-level FPP supplementation increased the serum superoxide dismutase activity and decreased the serum malondialdehyde level. Overall, replacing wheat flour with dietary FPP did not impair growth performance and improved intestinal digestive and absorptive functions, mucosal barrier integrity, and antioxidant capacity. Therefore, 4–6% may be considered a favorable dietary inclusion range for FPP in largemouth bass feed under the present experimental conditions. Because an unfermented pineapple pomace comparison group was not included, these effects should be interpreted as the effects of dietary FPP as a whole rather than as fermentation-specific effects.

## Simple Summary

Pineapple processing produces large amounts of leftover peel and pulp, while fish farming needs safe and sustainable feed ingredients. This study tested whether fermented pineapple pomace, a treated pineapple by-product, could be added to feed for young largemouth bass. The fish were fed diets containing different amounts of fermented pineapple pomace for ten weeks. The supplement did not affect fish growth or feed conversion. At suitable levels, especially 4 to 6 percent of the diet, it improved selected indicators of intestinal structure, digestive function, mucosal barrier integrity, and antioxidant capacity under controlled experimental conditions. These findings suggest that fermented pineapple pomace may be used as a partial replacement for wheat flour in largemouth bass diets. However, its economic feasibility and potential benefits under commercial farming or disease-challenge conditions require further evaluation.

## 1. Introduction

Pineapple, classified within the genus Ananas of the Bromeliaceae family, represents a major tropical fruit crop of considerable economic significance worldwide. With the rapid expansion of the pineapple processing industry, substantial quantities of pineapple pomace (PP) are generated during processing, accounting for approximately 50–60% of the whole fruit weight [1,2]. PP is rich in dietary fiber, pectin, soluble sugars, polyphenols, flavonoids, vitamins, minerals, and bromelain [3]. Among these components, bromelain is a cysteine protease with strong proteolytic activity, whereas polyphenols and flavonoids are generally associated with antioxidant and health-promoting properties [4]. These nutritional and bioactive characteristics suggest that PP may serve as a functional feed ingredient. However, PP is often discarded, stockpiled, or landfilled, which not only results in the underutilization of biomass resources but may also cause environmental pollution due to decomposition and organic matter accumulation. Accordingly, PP has potential as an alternative feed resource for the value-added utilization of agricultural by-products, although its nutritional and economic feasibility requires further evaluation.

Recently, PP and related processing by-products have attracted considerable interest as alternative feed resources for livestock and aquaculture species. Studies have reported that the dietary inclusion of PP can promote feed intake and improve nutrient utilization in ruminants, enhance growth performance and intestinal microbial balance in weaned piglets, and promote growth and improve health status in some aquatic animals, such as Pacific white shrimp (*Litopenaeus vannamei*) and Nile tilapia (*Oreochromis niloticus*) [5,6,7,8]. These findings indicate that PP may exert beneficial effects on animal growth and health, possibly by providing available nutrients, bioactive substances, and fermentable fiber substrates. Nevertheless, the direct application of PP in animal feeds is still limited by its relatively high fiber content, compact plant cell wall structure, high moisture content, and potential antinutritional effects [9]. Such characteristics can hinder nutrient liberation, reduce feed acceptability, and constrain the digestive utilization of PP, particularly in carnivorous fish that have poor capacity to metabolize complex carbohydrates and fiber-rich materials [10].

As a biological processing technique, fermentation can improve the applicability of unconventional feed ingredients by increasing nutrient availability and enhancing functional characteristics [11]. During fermentation, microorganisms and their enzymes can partially degrade cellulose, hemicellulose, pectin, and other complex structural carbohydrates, thereby disrupting plant cell wall components and facilitating the release of nutrients and bioactive compounds [12,13]. In addition, fermentation may produce organic acids, digestive enzymes, small peptides, and other microbial metabolites, which can improve feed palatability, enhance nutrient availability, modulate intestinal microbiota, and contribute to the host antioxidant defense system [14]. Fermentation may therefore modify the nutritional and functional properties of PP, providing a rationale for evaluating fermented pineapple pomace (FPP) as a candidate feed ingredient in aquafeeds. However, because the present feeding trial did not include an unfermented PP comparison group, the specific contribution of fermentation cannot be separated from the effects of PP itself.

Largemouth bass (*Micropterus salmoides*), also known as California bass, is an important freshwater aquaculture species due to its rapid growth, desirable flesh quality, absence of intermuscular bones, and high market value [15]. With the continued intensification of largemouth bass aquaculture, the demand for efficient, safe, and functional formulated feeds has increased. However, due to its carnivorous feeding habit, largemouth bass is particularly sensitive to dietary quality, nutrient availability, and the digestibility of feed ingredients. Therefore, assessing the effects of FPP on growth, intestinal health, and antioxidant capacity in largemouth bass is essential for evaluating its feasibility and functional value in aquafeeds. Based on these considerations, largemouth bass was selected as the experimental species to evaluate the practical effects of dietary FPP, used as a graded replacement for wheat flour, on growth performance, intestinal health, and antioxidant capacity. Because an unfermented PP comparison group was not included, the present study was designed to assess the feasibility of FPP as a functional aquafeed ingredient rather than to determine whether the observed responses were specifically attributable to fermentation. The findings were expected to provide preliminary information on the nutritional feasibility of using FPP as a partial replacement for wheat flour in largemouth bass diets under controlled experimental conditions.

## 2. Materials and Methods

### 2.1. Experimental Diets

The basal diet (FP0) was formulated with 10% wheat flour as the carbohydrate source. FPP was incorporated into the basal diet at inclusion levels of 2%, 4%, 6%, 8%, and 10% to replace equivalent proportions of wheat flour in the basal diet, and the corresponding diets were designated as FP2, FP4, FP6, FP8, and FP10, respectively. The PP used in this study was obtained commercially, whereas the FPP was prepared through a combined microbial–enzymatic fermentation process according to the supplier’s technical protocol, with the procedure proportionally scaled down according to the amount of PP used in the present experiment. The microbial inoculum, consisting of lactic acid bacteria, yeast, and Bacillus subtilis, and the enzyme preparations, mainly consisting of protease, mannanase, and cellulase, were supplied by Qingdao Vland Biotech Co., Ltd. (Qingdao, China). The recommended doses were approximately 1 g of microbial inoculum and 0.5 g of enzyme preparation per kg of raw material. The commercial microbial inoculum and enzyme preparations were used on a mass basis according to the technical protocol. The activity units of the individual enzyme preparations were not independently determined in the present study. The microbial composition was described according to the information available for the commercial inoculum; however, detailed strain-level identities and viable microbial counts were not determined. Briefly, water, brown sugar, and the microbial inoculum were thoroughly mixed and incubated anaerobically at 30–37 °C for 16–24 h to obtain the activated microbial suspension. The enzyme preparation was dissolved in water before use. Subsequently, PP, water, brown sugar, the activated microbial suspension, and the enzyme solution were thoroughly homogenized, with the moisture content of the inoculated material controlled at approximately 40%. The mixture was then transferred into one-way valve fermentation bags, sealed, and fermented anaerobically at 30–37 °C for more than 4 days. The pH of the fermented product was monitored after mixing the sample with distilled water at a ratio of 1:5, and fermentation was considered complete when the pH decreased to <5.0. This threshold was used only as a practical fermentation endpoint according to the technical protocol, and the exact terminal pH value was not systematically recorded as an analytical parameter. The organic acid profile of the final FPP was not determined. After fermentation, the FPP was immediately incorporated into the experimental diets. In the present study, the crude protein and crude lipid contents of unfermented PP were 6.31% and 0.90%, respectively, whereas those of FPP were 8.93% and 1.40%, respectively. The amino acid profiles of PP before and after microbial–enzymatic fermentation are presented in Table 1. The experimental diets were produced according to the procedure reported by [16]. After pelletization, the feeds were dried in a temperature-regulated chamber at 25 °C and then kept at −20 °C until further use. Details of the dietary formulation and proximate composition are provided in Table 2.

### 2.2. Experimental Fish

A ten-week feeding trial was conducted in a cement pond supplied with an aerated groundwater facility at Guangdong Ocean University. Juveniles were sourced from Helian Aquaculture Farm (Maoming, China). A total of 540 healthy juveniles with similar body size and an initial body weight of 8.89 ± 0.01 g were used in the feeding trial. After being fasted for 24 h, the fish were randomly allocated to 18 net cages measuring 1.2 m × 0.8 m × 1.0 m, with 30 individuals in each cage. Six dietary treatments were established, and each treatment contained three replicate cages. During the experiment, fish were hand-fed to apparent satiation two times per day at 07:30 and 17:30, and feed consumption was recorded daily. The water temperature ranged from 26 to 30 °C throughout the trial, while dissolved oxygen was maintained above 6.0 mg/L and ammonia nitrogen remained below 0.5 mg/L.

### 2.3. Sample Collection

After a 24 h fasting period, the biomass and number of fish in each cage were recorded for the subsequent assessment of growth performance. Twelve fish were randomly selected from each cage and anesthetized with a eugenol solution (Shanghai Yuanye Bio-Technology Co., Ltd., Shanghai, China) at a dilution ratio of 1:12,000 for the collection of blood and intestinal samples. Among these fish, four were used for morphological parameter assessment, including individual body weight, body length, visceral mass, liver weight, intestinal weight, and intestinal length. Foregut, hindgut, liver, and stomach tissues from these four fish were collected, immediately immersed in liquid nitrogen, and stored at −80 °C for the subsequent analyses of intestinal and hepatic enzyme activities. The remaining eight fish were used for hindgut tissue sampling. Among these eight fish, blood was first collected from six fish through the caudal vasculature. Blood samples from three fish were collected using syringes pretreated with sodium heparin (Shanghai Yuanye Bio-Technology Co., Ltd., Shanghai, China) for plasma preparation, whereas blood samples from the other three fish were collected using non-anticoagulant syringes for serum preparation. After blood collection, these six fish, together with the other two fish that were not used for blood collection, were dissected for hindgut tissue sampling. Hindgut samples from four fish were placed in EP tubes containing RNAlater solution (Wuhan Servicebio Technology Co., Ltd., Wuhan, China) for the quantitative analysis of intestinal gene expression. Hindgut samples from two additional fish were preserved in 4% paraformaldehyde for H&E staining, while those from the remaining two fish were immersed in 2.5% glutaraldehyde for transmission electron microscopy (TEM) examination.

### 2.4. Analysis

#### 2.4.1. Proximate Composition

The proximate composition of the experimental diets was analyzed following AOAC procedures [17]. Dry matter was determined by oven-drying the samples at 105 °C until a constant weight was reached. Crude protein was determined with a Dumas nitrogen analyzer (Primacs SN100; Skalar Analytical Instruments, Breda, The Netherlands), and the corresponding protein concentration was derived from nitrogen content using a conversion factor of 6.25. Crude lipid was quantified using the Soxhlet extraction method, with petroleum ether (boiling range: 30–60 °C; Xilong Scientific Co., Ltd., Shantou, China) used as the extraction solvent.

#### 2.4.2. Intestinal Digestive and Absorptive Enzyme Activities

Stomach pepsin activity was determined using a commercial assay kit obtained from Shanghai Enzyme-linked Biotechnology Co., Ltd. (Shanghai, China). Foregut amylase, lactase, lipase, trypsin, and alkaline phosphatase activities were determined using commercial assay kits. Amylase (A016-2-2) and alkaline phosphatase (A059-2-2) assay kits were supplied by Nanjing Jiancheng Bioengineering Institute (Nanjing, China), whereas lactase (ml092923), lipase (A054-2-1), and trypsin (ml036384) assay kits were obtained from Shanghai Enzyme-linked Biotechnology Co., Ltd. (Shanghai, China).

#### 2.4.3. Intestinal Tissue Sections

For histological examination, hindgut samples preserved in 4% paraformaldehyde were processed using increasing ethanol concentrations, cleared with xylene, and embedded in paraffin to maintain tissue structure and enable subsequent sectioning. The solidified paraffin blocks were subsequently sectioned into 5 μm-thick sections using a pathological microtome (RM2016, Leica Microsystems GmbH, Wetzlar, Germany). The sections were then stained with H&E. The prepared sections were examined and photographed under an inverted fluorescence microscope (Eclipse Ti-E, Nikon, Tokyo, Japan). Six fields were randomly selected from each transverse section for measuring the villus height, villus width, and muscularis thickness.

For TEM analysis, hindgut samples fixed in 2.5% glutaraldehyde were washed three times with 0.1 mol/L phosphate buffer (pH 7.4), with each wash lasting 15 min. The samples were subsequently post-fixed in 1% osmium tetroxide prepared in phosphate-buffered saline for 2 h at room temperature in the dark, and then rinsed three more times with 0.1 mol/L phosphate buffer. Afterward, the tissues were dehydrated using a graded ethanol series, followed by two additional dehydration steps in 100% acetone. The intestinal tissues were subsequently embedded in resin using an embedding system, incubated overnight at 37 °C, and then polymerized at 65 °C for more than 48 h on the following day. After trimming, the resin-embedded blocks were cut into 60 nm ultrathin sections with a Leica UC7 ultramicrotome (Leica, Wetzlar, Germany), and the sections were subsequently mounted onto Formvar-coated 150-mesh copper grids. The grids were incubated with ethanolic uranyl acetate at a concentration of 2% for 8 min in darkness, and were subsequently rinsed three times with 70% ethanol, followed by three additional washes using ultrapure water. The sections were subsequently stained with 2.6% lead citrate for 8 min under carbon dioxide-free conditions, rinsed three times with ultrapure water, and gently dried with filter paper to eliminate residual liquid from the grid surface. Finally, the grids were transferred to a grid storage box and dried overnight at room temperature. The ultrathin sections were observed and photographed using TEM (HT7800, Hitachi, Tokyo, Japan).

#### 2.4.4. Intestinal Mucosal Permeability Parameters

For the plasma samples, assay kits supplied by Shanghai Enzyme-linked Biotechnology Co., Ltd. (Shanghai, China) were used to quantify endothelin-1 (ET-1; ml955101), D-lactate (D-LA; ml950994), lipopolysaccharide (LPS; ML505648), and diamine oxidase (DAO; ml511201).

#### 2.4.5. Activities of Enzymes Related to Intestinal Mucosal Barrier Function

Commercial assay kits for intestinal lysozyme (LZM; ml905258), mucin 2 (MUC2; ml951514), immunoglobulin M (IgM; ml326413), secretory immunoglobulin T (sIgT; ml204789), interleukin-1β (IL-1β; ml002302), interleukin-8 (IL-8; ml995241), tumor necrosis factor-α (TNF-α; ml002095), interleukin-10 (IL-10; ml980277), and transforming growth factor-β (TGF-β; ml107101) were obtained from Shanghai Enzyme-linked Biotechnology Co., Ltd. (Shanghai, China).

#### 2.4.6. Antioxidant Enzyme Activities

Serum samples were used to determine superoxide dismutase (SOD), catalase (CAT), reduced glutathione (GSH), total antioxidant capacity (T-AOC), and malondialdehyde (MDA). Intestinal and liver tissue samples were thawed on ice, weighed, and homogenized with ice-cold physiological saline at a ratio of 1:9 (*w*/*v*) to prepare 10% tissue homogenates. The homogenates were then centrifuged at 3500 rpm for 10 min at 4 °C, and the supernatants were collected for the determination of SOD, CAT, peroxidase (POD), T-AOC, and MDA. Commercial assay kits for SOD (A001-3-2), CAT (A007-1-1), GSH (A006-2-1), T-AOC (A015-2-1), and MDA (A003-1-2) were supplied by Nanjing Jiancheng Bioengineering Institute (Nanjing, China). The assay kit for POD (ml321110) was obtained from Shanghai Enzyme-linked Biotechnology Co., Ltd. (Shanghai, China). All assays were performed according to the manufacturers’ instructions.

#### 2.4.7. Real-Time Quantitative PCR Analysis of RNA

Total RNA was obtained from hindgut samples using the TransZol UP Plus RNA Kit from TransGen Biotech Co., Ltd. (Beijing, China), in accordance with the manufacturer’s protocol. RNA integrity was checked by 1% agarose gel electrophoresis, while purity and concentration were assessed with a NanoDrop 2000 spectrophotometer (Thermo Scientific, Waltham, MA, USA). First-strand complementary DNA (cDNA) was generated with a reverse transcription kit from Aidlab Biotechnologies Co., Ltd. (Beijing, China) and then maintained at −20 °C until use. The primer pairs applied for qRT-PCR analysis were produced by Shanghai Biotechnology Co., Ltd. (Shanghai, China), and the corresponding sequences were adopted from previously published studies, as shown in Table 3. qRT-PCR was performed according to the manufacturer’s instructions using the SYBR^®^ Premix Pro HS qPCR Kit II (Aidlab Biotechnologies Co., Ltd., Beijing, China). Each qRT-PCR reaction mixture had a final volume of 10 μL. *β-Actin* served as the internal control, and the abundance of target gene transcripts was determined according to the 2^−ΔΔCt^ approach.

#### 2.4.8. Calculations and Statistical Analysis

Data are expressed as the mean ± standard error of the mean (SEM). The cage was considered the experimental unit for statistical analysis, with three replicate cages per treatment (*n* = 3). Following confirmation that the data met the assumptions of normality and variance homogeneity, one-way analysis of variance (ANOVA) was performed for statistical comparison. Differences among treatment means were further evaluated using Duncan’s multiple range test. Statistical significance was set at *p* < 0.05. All analyses were conducted with SPSS 26.0 for Windows (SPSS Inc., Chicago, IL, USA).

## 3. Results

### 3.1. Growth Performance

Dietary FPP level did not significantly affect the final body weight (FBW), weight gain rate (WGR), specific growth rate (SGR), feeding rate (FR), feed conversion ratio (FCR), protein efficiency ratio (PER), survival rate (SR), condition factor (CF), or intestosomatic index (ISI) in largemouth bass (*p* > 0.05; Table 4). Compared with the control group, the viscerosomatic (VSI) and hepatosomatic index (HSI) were significantly increased in the FP10 group (*p* < 0.05).

### 3.2. Intestinal Digestion and Absorption Function

Dietary FPP level did not significantly affect intestinal muscularis thickness or pepsin activity in largemouth bass (*p* > 0.05; Table 5 and Table 6). Compared with the control group, villus height was significantly increased in the FP4 and FP6 groups, whereas villus width was significantly increased in the FP4, FP6, and FP8 groups (*p* < 0.05). In addition, amylase activity was significantly increased in the FP2, FP4, and FP6 groups; lactase activity was significantly increased in the FP6 and FP10 groups; and lipase activity was significantly increased in all FPP-supplemented groups (*p* < 0.05). Trypsin activity was significantly increased in the FP4, FP6, FP8, and FP10 groups, while alkaline phosphatase activity was significantly increased in the FP6 and FP10 groups compared with the control group (*p* < 0.05).

### 3.3. Intestinal Morphology

The intestinal architecture remained intact across all experimental groups, including those with different inclusion levels of FPP. The intestinal villi were regularly arranged, and the epithelial cells exhibited normal morphology, with no obvious disruption of tissue structure, inflammatory cell infiltration, or pathological alterations. These findings indicate that FPP did not exert adverse effects on the intestinal morphology of largemouth bass within the tested inclusion range (Figure 1). In addition, ultrastructural observations of the intestinal epithelium showed that the FP6 group exhibited improved tight junction integrity and a more compact arrangement of intestinal microvilli (Figure 2).

### 3.4. Intestinal Mucosal Barrier Function

Compared with the control group, ET-1 level, DAO activity, and LPS level were significantly decreased in all FPP-supplemented groups (*p* < 0.05; Table 7). The D-LA level was significantly reduced in the FP2 group but significantly increased in the FP8 group compared with the control group (*p* < 0.05). For intestinal tight junction-related genes, *Claudin-4* expression was significantly elevated in the FP4 group compared with the control group (*p* < 0.05; Figure 3). *ZO-1* and *Occludin* expressions were significantly upregulated in the FP6 group, whereas *Claudin-1* expression showed a significant increase in the FP10 group (*p* < 0.05). Dietary FPP supplementation had no significant influence on the TGF-β level (*p* > 0.05). Compared with the control group, LZM activity was significantly enhanced in the FP4–FP10 groups (*p* < 0.05; Table 8). MUC2 level was significantly increased in the FP4, FP6, FP8, and FP10 groups. IgM level showed a significant elevation in the FP8 group, while sIgT level was significantly increased in the FP4, FP6, FP8, and FP10 groups (*p* < 0.05). The IL-1β level was significantly reduced in the FP6, FP8, and FP10 groups, whereas the IL-8 level was significantly decreased only in the FP2 group (*p* < 0.05). The TNF-α level was significantly reduced in the FP2–FP8 groups. IL-10 level was significantly decreased in the FP2 group but significantly increased in the FP4, FP6, and FP10 groups compared with the control group (*p* < 0.05). For intestinal inflammatory cytokine-related genes, dietary FPP supplementation did not significantly influence *IL-1β* expression (*p* > 0.05). Compared with the control group, *IL-8* expression was significantly downregulated in the FP4 group, *IL-10* expression was significantly upregulated in the FP6 and FP8 groups, and *TGF-β* expression showed a significant elevation in the FP8 group (*p* < 0.05; Figure 4).

### 3.5. Antioxidant Capacity

Compared with the control group, serum SOD activity was significantly increased in the FP2 and FP4 groups, whereas serum GSH level was significantly decreased in the FP2 group (*p* < 0.05; Table 9). Serum CAT activity was significantly increased in all FPP-supplemented groups. Serum T-AOC was significantly increased in the FP4, FP6, and FP8 groups, while serum MDA level was significantly decreased in the FP2 and FP4 groups compared with the control group (*p* < 0.05). Dietary FPP supplementation had no significant effect on intestinal SOD activity (*p* > 0.05). Compared with the control group, intestinal CAT activity was significantly enhanced in the FP4, FP6, FP8, and FP10 groups. Intestinal POD activity was significantly increased in the FP4–FP10 groups, and intestinal T-AOC was significantly increased in the FP6–FP10 groups (*p* < 0.05). Intestinal MDA level was significantly reduced in the FP4, FP6, FP8, and FP10 groups compared with the control group (*p* < 0.05). Dietary FPP supplementation did not significantly influence the liver SOD activity (*p* > 0.05). Compared with the control group, liver CAT activity was significantly increased in the FP10 group, whereas liver POD activity was significantly decreased in the FP2 group (*p* < 0.05). Liver T-AOC was significantly increased in the FP2, FP4, FP6, and FP8 groups. Liver MDA level was significantly decreased in the FP2, FP4, and FP6 groups but significantly increased in the FP10 group compared with the control group (*p* < 0.05).

## 4. Discussion

In the present study, replacing wheat flour with 0–10% FPP did not significantly affect growth performance or feed conversion in largemouth bass, indicating that dietary FPP could be used as a partial replacement for wheat flour without impairing growth under the present experimental conditions. Pineapple pomace has been reported to contain pectin; however, the pectin content and degree of esterification of the PP and FPP used in this study were not determined. Therefore, the potential role of pectin characteristics in the growth response could not be further evaluated. The numerical increases in VSI and HSI in the FP8 group and their significant increases in the FP10 group may indicate altered visceral nutrient deposition or hepatic metabolism at high FPP inclusion levels. However, further histological and biochemical analyses are required to clarify their physiological significance. Although FPP did not produce a growth-enhancing effect in the present study, it did not negatively affect feed intake, feed conversion, or overall growth status. This result may be related to the carnivorous feeding habit of largemouth bass, its limited ability to utilize complex carbohydrates and fibrous ingredients, and the use of FPP as a partial replacement for wheat flour rather than as an additional nutrient source. It should be noted that FPP was used to replace wheat flour on an equal-weight basis in the present diet formulation. Therefore, the dietary treatments involved not only increasing FPP inclusion but also a concurrent reduction in wheat flour level. Although the experimental diets were formulated to contain similar crude protein and crude lipid levels, this replacement may have altered dietary starch availability, carbohydrate composition, fiber content, residual sugars, fermentation-derived components, organic acids, and microbial residues. Therefore, the present results indicate that replacing wheat flour with FPP maintained growth performance while improving several indicators of intestinal digestive function and barrier status in largemouth bass. These findings support the nutritional feasibility of FPP as a partial wheat flour replacement under the present experimental conditions. Nevertheless, because FPP was used to replace wheat flour on an equal-weight basis, the observed responses should be interpreted as the overall effect of dietary replacement with FPP rather than the effect of a single dietary component. Despite these simultaneous dietary changes, the absence of growth depression may be partly associated with the overall nutritional and functional properties of FPP. Fermentation has been reported to modify fibrous plant by-products by partially degrading structural carbohydrates, disrupting the plant cell wall matrix, and promoting the release of encapsulated nutrients and bioactive compounds [12,21]. Moreover, microbial activity may further enhance substrate quality by promoting the formation of organic acids, endogenous enzymes, small peptides, and other bioactive metabolites [22]. These changes could contribute to improved palatability, nutrient accessibility, and digestive utilization, thereby offsetting the limitations associated with the direct use of fibrous plant by-products [23]. However, because an unfermented PP group was not included in the present study, these possible fermentation-related changes cannot be confirmed as the direct cause of the observed responses. Therefore, the present findings should be interpreted as the practical effects of dietary FPP replacement rather than as direct evidence of fermentation-specific benefits. From the perspective of feed resource utilization, the application of FPP may facilitate the partial replacement of conventional feed ingredients and promote the high-value utilization of PP as a feed resource. Nevertheless, the absence of a growth-promoting effect suggests that the functional value of FPP may be more prominently reflected in intestinal health, barrier function, and antioxidant status rather than in direct growth enhancement.

Intestinal health is fundamental to nutrient utilization and overall physiological status in fish, and efficient digestive and absorptive capacity is one of its core components [24]. In the present study, dietary supplementation with FPP had no significant effect on intestinal muscularis thickness or pepsin activity in largemouth bass. However, the FP4 and FP6 diets increased the villus height, whereas the FP4, FP6, and FP8 diets increased the villus width. Dietary FPP also enhanced several intestinal digestive and absorptive enzyme activities, but the responsive inclusion levels differed among enzymes. Collectively, these findings suggest that dietary FPP replacement was associated with improved intestinal digestive and absorptive status in largemouth bass. For example, dietary pineapple waste crude extract increased the microvilli volume in the hindgut of Pacific white shrimp, although protease and trypsin activities decreased at the highest supplementation level [6]. In rainbow trout, apple pomace-derived pectin significantly increased the protease and amylase activities, while lipase activity was not significantly affected [25]. Similarly, dietary pectin at 8–16% increased foregut trypsin and amylase activities in juvenile rainbow trout, although sucrase activity in the foregut and midgut was reduced. These findings indicate that fruit by-products and pectin-rich ingredients can regulate intestinal structure and digestive enzyme profiles, but their effects vary depending on species, ingredient source, inclusion level, and enzyme category. The beneficial responses observed in fish fed FPP may be associated with the combined effects of FPP inclusion and wheat flour replacement, including changes in nutrient availability, fiber and pectin content, residual sugars, and fermentation-derived components. Therefore, the increased villus height or width and enhanced digestive enzyme activities observed in the present study suggest that FPP mainly improved the intestinal functional status of largemouth bass rather than directly promoting growth. Such improvement in digestive and absorptive function may also provide a physiological basis for the subsequent enhancement of intestinal barrier integrity. Thus, the intestinal morphological and enzymatic responses should be viewed as coordinated improvements in digestive and absorptive capacity rather than as isolated changes in individual parameters.

The intestinal mucosal barrier forms a crucial boundary between the intestinal lumen and the host internal environment, and its integrity is essential for preventing the translocation of luminal toxins and pathogens [26]. Impairment of barrier function increases intestinal permeability, allowing luminal substances to enter the circulation and trigger local or systemic inflammation and metabolic disturbances. ET-1, LPS, DAO, and D-LA are key biomarkers for evaluating intestinal mucosal permeability and injury [27]. In the present study, dietary FPP reduced the plasma endothelin-1 level, diamine oxidase activity, and lipopolysaccharide level in all supplemented groups, whereas the D-lactate level was decreased in the FP2 group but increased in the FP8 group. Overall, the decreases in ET-1, LPS, and DAO suggest that dietary FPP improved the intestinal mucosal barrier status and reduced permeability-related injury in largemouth bass, although D-LA responded differently among inclusion levels. As a fiber-rich fermented ingredient, FPP may act mainly within the intestinal lumen by modifying the local intestinal environment and fermentation-related microbial metabolites. However, organic acids, polyphenols, bromelain activity, pectin characteristics, intestinal microbiota, and short-chain fatty acids were not measured in the present study. Therefore, the specific mechanisms underlying the observed changes in intestinal permeability remain to be further clarified. Intestinal epithelial cells, together with the tight junction proteins linking adjacent cells, constitute the major structural basis of the mechanical barrier. Among these proteins, *ZO-1*, *Occludin*, *Claudin-1*, and *Claudin-4* play indispensable roles in sustaining barrier integrity. Their expression levels are key indicators of barrier function, and impairment is often associated with their downregulation or abnormal distribution [28]. In the present study, dietary FPP increased the expression of several tight junction-related genes and improved epithelial ultrastructure, as shown by the more intact tight junctions and compact microvilli arrangement observed in the FP6 group. Together with the reductions in permeability-related biomarkers, these findings suggest that FPP supported intestinal mechanical barrier integrity in largemouth bass. As a fiber-rich fermented ingredient, FPP may influence epithelial barrier function mainly through changes in the local intestinal luminal environment. Previous studies have shown that short-chain fatty acids, particularly acetate and butyrate, can enhance intestinal barrier integrity and upregulate tight junction-related genes in fish [29,30]. Therefore, the tight junction-related responses observed in this study may be partly associated with fermentation-related metabolites. However, because intestinal microbiota and short-chain fatty acids were not measured, this possible pathway requires further verification. The chemical and immune barriers of fish act synergistically as important lines of defense against pathogens and environmental stressors [31]. The chemical barrier primarily consists of the mucus layer secreted by epithelial cells, which contain nonspecific bactericidal or bacteriostatic substances. This layer, mainly composed of mucins secreted by goblet cells, prevents direct contact between pathogens and the intestinal epithelium [32]. Meanwhile, secretory molecules such as lysozyme and antimicrobial peptides further enhance local antibacterial capacity. Dietary supplementation with FPP increased the intestinal mucin 2 levels and lysozyme activity at appropriate inclusion levels, indicating that FPP supplementation was associated with enhanced antibacterial capacity and strengthened intestinal chemical barrier function by stabilizing the mucus layer. These effects may be related to the overall composition of FPP; however, the specific components responsible for the increased MUC2 level and lysozyme activity were not determined in the present study. The immune barrier is another important component of intestinal mucosal defense in fish, in which immunoglobulins play key roles in pathogen recognition and neutralization. IgM is the predominant antibody involved in systemic humoral immunity, whereas sIgT is generally considered a mucosal immunity-related immunoglobulin that contributes to local defense at mucosal surfaces [33]. In the present study, dietary FPP increased intestinal secretory immunoglobulin T levels in the FP4, FP6, FP8, and FP10 groups and elevated immunoglobulin M level in the FP8 group, suggesting that FPP enhanced mucosal immune defense in largemouth bass without inducing immunosuppression. Comparable immunomodulatory effects have also been observed in fish receiving pectin-derived functional ingredients; for instance, apple pomace-derived pectin elevated serum lysozyme activity, complement activity, and the total immunoglobulin levels in rainbow trout [25], while orange peel-derived pectin improved the serum and skin mucus immune parameters, including total immunoglobulin and lysozyme activity, in common carp [20]. The increase in secretory immunoglobulin T may be particularly relevant to the improved mucosal barrier status observed in this study, because secretory immunoglobulin T contributes to immune exclusion by limiting pathogen adhesion and colonization at mucosal surfaces. Meanwhile, the moderate increase in IgM suggests that FPP may also support humoral immune defense. Therefore, the changes in intestinal immunoglobulins further indicate that appropriate FPP supplementation strengthened the immune component of the intestinal barrier. Cytokines play central roles in regulating intestinal immune homeostasis by coordinating pro-inflammatory and anti-inflammatory responses. When pathogens breach the barrier, pro-inflammatory cytokines are rapidly produced to recruit and activate more immune cells for pathogen clearance [34]. However, excessive production can induce inflammatory injury, disrupt tissue barriers, and even lead to cell death. In contrast, anti-inflammatory cytokines suppress excessive immune activation and promote inflammation resolution and tissue repair [35]. In this study, dietary FPP modulated intestinal cytokine profiles in a dose-dependent manner, generally reducing selected pro-inflammatory cytokines and enhancing anti-inflammatory responses at appropriate inclusion levels. The rebound of TNF-α at FP10 suggests that its anti-inflammatory response was non-linear and may have been attenuated at excessive FPP inclusion, whereas the sustained reductions in ET-1 and DAO indicate that inflammatory signaling and mucosal permeability did not respond identically. Consistent with these findings, fermented Astragalus has been reported to mitigate DSS-induced colitis by suppressing pro-inflammatory cytokines and enhancing anti-inflammatory mediators, while microbiota-derived short-chain fatty acids may further support intestinal immune homeostasis by promoting *IL-10* production in T cells [36,37]. These cytokine responses may be associated with the overall dietary replacement with FPP and changes in the intestinal luminal environment. However, because intestinal microbiota, short-chain fatty acids, and specific bioactive components were not determined, the mechanisms underlying these immune responses require further investigation. Taken together, the coordinated changes in permeability-related biomarkers, tight junction-related gene expression, epithelial ultrastructure, mucus-related factors, immunoglobulins, and cytokines indicate that dietary FPP supported intestinal mucosal barrier function at multiple levels.

Antioxidant defense is essential for maintaining physiological health, coping with environmental stress, and ensuring stable aquaculture production in fish [38]. In the present study, dietary supplementation with FPP enhanced T-AOC in the liver and intestine at specific inclusion levels, which may be partly associated with changes in antioxidant enzyme activities. Similar results have been reported in juvenile largemouth bass fed fermented tea residue, which increased hepatic SOD, GSH-Px, CAT activities and T-AOC while reducing MDA content [39]. In addition, dietary *Lentinus edodes* fermentation supplementation enhanced hepatic CAT activity and decreased hepatic MDA level in largemouth bass fed high plant protein diets [40]. These studies provide relevant context for fermented plant-derived feed ingredients; however, their mechanisms should not be directly generalized to FPP because fermentation substrates and bioactive profiles differ among products. In the present study, the antioxidant response to FPP was reflected mainly by increased T-AOC and CAT activity at appropriate inclusion levels and reduced MDA level at low inclusion levels. Because fermentation-derived components such as polyphenols, peptides, and organic acids were not characterized in the FPP used here, the specific components responsible for these antioxidant responses remain unclear. Hydrogen peroxide acts as an important redox signaling molecule under physiological conditions; however, excessive accumulation can cause oxidative damage and cytotoxicity [41]. The increase in liver CAT activity induced by FPP may represent an adaptive response to prevent hydrogen peroxide accumulation and maintain oxidative balance. Oxidative disturbances in key organs such as the liver and intestine may influence circulating oxidative biomarkers and systemic antioxidant status. This process may elevate systemic oxidative stress biomarkers, reduce T-AOC, and contribute to systemic inflammation or even multiple-organ dysfunction [42]. Accordingly, changes in circulating antioxidant and oxidative stress biomarkers may provide information on systemic redox status [43]. In this study, FPP increased the serum CAT activity and T-AOC at appropriate inclusion levels, indicating improved systemic antioxidant defense. Additionally, low-dose supplementation increased the serum SOD activity and reduced the MDA level, suggesting effective alleviation of oxidative stress. However, the increased hepatic MDA level in the FP10 group suggests that excessive FPP supplementation may attenuate its antioxidant benefits and could increase hepatic lipid peroxidation at the highest inclusion level. Notably, hepatic MDA level showed a similar trend, first decreasing and then increasing, consistent with serum MDA changes. This suggests that alterations in local organ oxidative status may ultimately be reflected at the systemic level.

Several limitations of the present study should be acknowledged. First, an unfermented PP group was not included. Therefore, the experimental design did not allow us to distinguish whether the observed responses were attributable to PP itself, the reduction in wheat flour, fermentation-induced changes, organic acids, microbial metabolites, microbial residues, or their combined effects. Second, the fermentation process and the resulting FPP were not comprehensively characterized. Specifically, the activity units of the enzyme preparations were not independently determined, the exact final pH was not systematically recorded beyond confirmation that the practical fermentation endpoint of pH < 5.0 had been reached, and organic acid profiles, viable microbial counts, and detailed strain-level information were not determined. The absence of these data limits a more detailed interpretation of the biochemical and microbial changes occurring during fermentation and limits our ability to link the observed physiological responses to specific fermentation-derived characteristics. In addition, crude fiber and nitrogen-free extract were not determined in the experimental diets, which limits the quantitative assessment of changes in dietary fiber and carbohydrate fractions caused by the equal-weight replacement of wheat flour with FPP. Accordingly, the present findings demonstrate the practical effects of dietary FPP as a replacement for wheat flour, but they do not isolate the specific contribution of fermentation or fully separate the effects of FPP inclusion from those associated with wheat flour reduction and related changes in dietary carbohydrate composition. Future studies should include both unfermented PP and FPP groups, provide more comprehensive physicochemical and microbial characterization of the fermentation process and the resulting fermented product, and use diets with better characterized fiber and carbohydrate fractions to clarify the mechanisms underlying the effects of FPP.

## 5. Conclusions

Dietary supplementation with 0–10% FPP did not impair the growth performance of largemouth bass. Under the present experimental conditions, appropriate FPP inclusion levels, particularly 4–6%, were associated with improved intestinal digestive and absorptive function, enhanced mucosal barrier integrity, and strengthened antioxidant capacity. Therefore, 4–6% FPP may be considered a favorable dietary inclusion range for largemouth bass under the present experimental conditions. However, because an unfermented PP group was not included, these effects should be interpreted as the effects of dietary FPP as a whole rather than as fermentation-specific effects. Further studies comparing unfermented PP and FPP are needed to determine the specific contribution of fermentation.

## Figures and Tables

**Figure 1:**
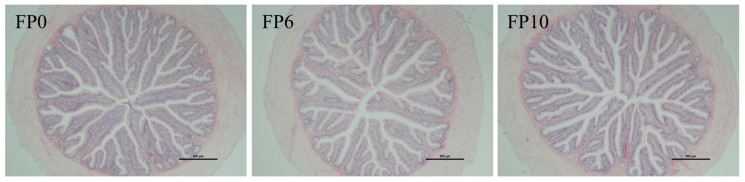
Effects of dietary fermented pineapple pomace level on the intestinal histomorphology in largemouth bass (100×; H&E).

**Figure 2:**
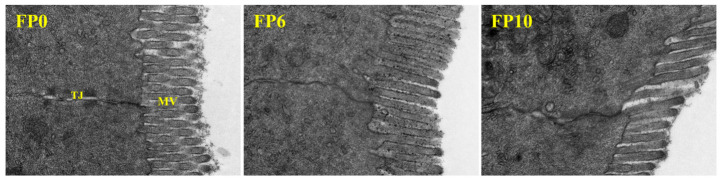
Effects of dietary fermented pineapple pomace on the intestinal epithelial ultrastructure in largemouth bass (TEM; 5000×). TJ, tight junctions; MV, microvilli.

**Figure 3:**
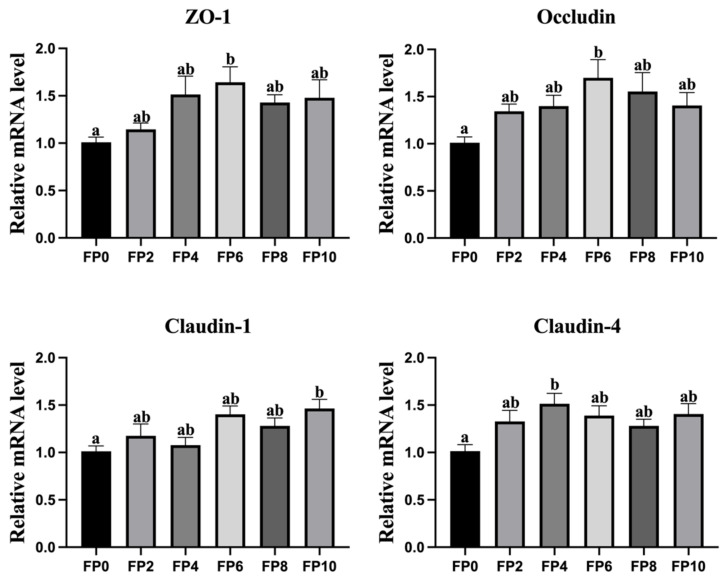
Effect of dietary fermented pineapple pomace inclusion on the relative expression levels of tight junction-related genes in the hindgut of largemouth bass. Values are means with their standard errors represented by vertical bars (*n* = 3). ^a,b^ Means with different letters indicate significant difference (*p* < 0.05). *ZO-1*, zonula occludens protein 1.

**Figure 4:**
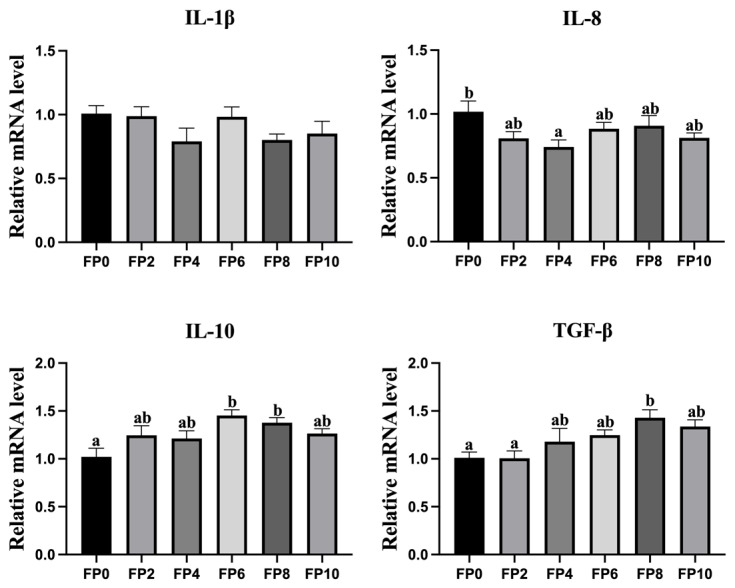
Effect of dietary fermented pineapple pomace inclusion on the relative expression levels of inflammation-related genes in the hindgut of largemouth bass. Values are means with their standard errors represented by vertical bars (*n* = 3). ^a,b^ Means with different letters indicate significant difference (*p* < 0.05). *IL-1β*, interleukin-1 beta; *IL-8*, interleukin-8; *IL-10*, interleukin-10; *TGF-β*, transforming growth factor-beta.

**Table 1:** Nutrient composition of pineapple pomace before and after enzyme-catalyzed co-fermentation (%, dry matter basis).

Ingredient	Pineapple Pomace	Fermented Pineapple Pomace
Crude protein	6.31	8.93
Crude lipid	0.90	1.40
Acid-soluble protein	3.43	3.74
Aspartic acid	0.37	0.58
Threonine	0.12	0.20
Serine	0.15	0.26
Glutamic acid	0.38	0.87
Glycine	0.17	0.33
Alanine	0.18	0.30
Cystine	0.06	0.03
Valine	0.16	0.25
Methionine	0.03	0.05
Isoleucine	0.13	0.19
Leucine	0.18	0.34
Tyrosine	0.08	0.12
Phenylalanine	0.14	0.25
Lysine	0.07	0.09
Histidine	0.05	0.11
Arginine	0.10	0.21
Proline	0.15	0.25
ΣFree amino acids	2.51	4.41

**Table 2:** Experimental feed formulation and proximate composition (%, dry matter basis).

	FP0	FP2	FP4	FP6	FP8	FP10
Fish meal	24.00	24.00	24.00	24.00	24.00	24.00
Chicken meal	7.00	7.00	7.00	7.00	7.00	7.00
Cottonseed protein concentrate	15.00	15.00	15.00	15.00	15.00	15.00
Soybean meal	31.80	31.80	31.80	31.80	31.80	31.80
Wheat flour	10.00	8.00	6.00	4.00	2.00	0.00
Fermented pineapple pomace	0.00	2.00	4.00	6.00	8.00	10.00
Fish oil	3.50	3.50	3.50	3.50	3.50	3.50
Soybean oil	4.00	4.00	4.00	4.00	4.00	4.00
Soybean lecithin	1.50	1.50	1.50	1.50	1.50	1.50
Vitamin premix ^a^	0.30	0.30	0.30	0.30	0.30	0.30
Mineral premix ^b^	0.70	0.70	0.70	0.70	0.70	0.70
Others ^c^	2.20	2.20	2.20	2.20	2.20	2.20
Proximate composition						
Dry matter (DM, %)	86.66	86.96	85.72	86.68	86.43	85.23
Crude protein (% DM)	49.44	49.41	49.46	49.53	49.52	49.59
Crude lipid (% DM)	11.19	11.17	11.21	11.16	11.14	11.20

^a^ Vitamin premix (g/kg of mixture) included 1.35 g retinyl acetate, 0.024 g cholecalciferol, 40 g DL-α-tocopheryl acetate, 4 g menadione, 8.5 g thiamine hydrochloride, 9 g riboflavin, 11 g pyridoxine hydrochloride, 0.014 g vitamin B12, 40 g ascorbic acid, 18 g calcium D-pantothenate, 2.4 g folic acid, 0.225 g D-biotin, and 75 g inositol. ^b^ Mineral premix (g/kg of mixture) included 186 g FeSO_4_·7H_2_O, 53 g ZnSO_4_·7H_2_O, 25 g MnSO_4_·H_2_O, 2.7 g CuSO_4_·5H_2_O, 0.4 g CoCl_2_·6H_2_O, 0.1 g Na_2_SeO_3_, and 0.13 g KI. ^c^ Others included 1.5% Ca(H_2_PO_4_)2, 0.2% sodium chloride, 0.4% choline chloride, 0.05% yttrium oxide, 0.02% vitamin C, and 0.03% ethoxyquin.

**Table 3:** Primer sequence used for qPCR analysis.

Gene	Primer Sequence (5′–3′)	Accession No.	Amplification Efficiency	Source
*β-actin*	F: GGACACGGAAAGGATTGACAG R: CGGAGTCTCGTTCGTTATCGG	XM_038695351.1	94.73%	[18]
*Claudin-1*	F: CCAGGGAAGGGGAGCAATG R: GCTCTTTGAACCAGTGCGAC	XM_038713307.1	96.32	[19]
*Claudin-4*	F: TAATCGCTATGGTGGGAGCC R: GCCCCGATCTCCATCTTCTG	XM_038708626.1	90.05%	[18]
*IL-1β*	F: CGTGACTGACAGCAAAAAGAGG R: GATGCCCAGAGCCACAGTTC	XM_038696252.1	104.21%	[18]
*IL-8*	F: CGTTGAACAGACTGGGAGAGATG R: AGTGGGATGGCTTCATTATCTTGT	XM_038704088.1	108.93%	[18]
*IL-10*	F: CGGCACAGAAATCCCAGAGC R: CAGCAGGCTCACAAAATAAACATCT	XM_038696252.1	108.32%	[18]
*Occludin*	F: GATATGGTGGCAGCTACGGT R: TCCTACTGCGGACAGTGTTG	XM_005472621.4	92.53%	[20]
*TGF-β*	F: GCTCAAAGAGAGCGAGGATG R: TCCTCTACCATTCGCAATCC	XM_038693206.1	96.82%	[18]
*ZO-1*	F: ATCTCAGCAGGGATTCGACG R: CTTTTGCGGTGGCGTTGG	XM_038701018.1	90.91%	[19]

*β-actin*, beta-actin; *IL-1β*, interleukin-1 beta; *IL-8*, interleukin-8; *IL-10*, interleukin-10; *TGF-β*, transforming growth factor-beta; *ZO-1*, zonula occludens protein 1.

**Table 4:** Effects of dietary fermented pineapple pomace inclusion level on growth performance and somatic indices in largemouth bass.

	FP0	FP2	FP4	FP6	FP8	FP10	*p* Value
IBW (g)	8.91 ± 0.02	8.91 ± 0.02	8.89 ± 0.02	8.90 ± 0.01	8.84 ± 0.02	8.91 ± 0.02	0.207
FBW (g)	66.05 ± 1.37	65.46 ± 0.18	65.35 ± 0.46	64.50 ± 1.07	64.25 ± 0.62	64.32 ± 0.96	0.628
WGR (%)	641.46 ± 16.43	634.85 ± 3.73	635.14 ± 5.63	624.88 ± 11.40	622.63 ± 11.24	614.20 ± 15.90	0.613
SGR (%/d)	2.86 ± 0.03	2.85 ± 0.01	2.85 ± 0.01	2.83 ± 0.02	2.83 ± 0.02	2.81 ± 0.03	0.604
FR (%/d)	2.05 ± 0.05	2.05 ± 0.05	2.11 ± 0.04	2.13 ± 0.02	2.14 ± 0.05	2.13 ± 0.04	0.451
FCR	0.94 ± 0.03	0.94 ± 0.02	0.97 ± 0.02	0.98 ± 0.01	0.99 ± 0.03	0.99 ± 0.02	0.482
PER (%)	2.16 ± 0.07	2.15 ± 0.05	2.09 ± 0.04	2.05 ± 0.03	2.04 ± 0.06	2.04 ± 0.05	0.338
SR (%)	98.89 ± 1.11	98.89 ± 1.11	96.67 ± 1.92	100.00 ± 0.00	97.78 ± 1.11	97.78 ± 2.22	0.660
CF (g/cm^3^)	2.34 ± 0.07	2.32 ± 0.04	2.35 ± 0.04	2.35 ± 0.03	2.38 ± 0.02	2.43 ± 0.03	0.518
VSI (%)	6.67 ± 0.06 ^a^	6.68 ± 0.11 ^a^	6.60 ± 0.10 ^a^	6.77 ± 0.13 ^a^	6.86 ± 0.06 ^ab^	7.10 ± 0.10 ^b^	0.032
HSI (%)	0.72 ± 0.02 ^ab^	0.70 ± 0.04 ^a^	0.68 ± 0.03 ^a^	0.75 ± 0.03 ^ab^	0.89 ± 0.05 ^bc^	0.95 ± 0.10 ^c^	0.015
ISI (%)	0.85 ± 0.04	0.90 ± 0.02	0.88 ± 0.01	0.85 ± 0.03	0.86 ± 0.01	0.87 ± 0.06	0.872

Values are expressed as means ± standard error of the means (*n* = 3). Means within the same row with different superscripts indicate statistically significant differences among treatments (*p* < 0.05). IBW, initial body weight; FBW, final body weight; WGR (weight gain rate) = 100 × (final body weight [g] − initial body weight [g])/initial body weight [g]; SGR (specific growth rate) = 100 × [ln(final body weight) − ln(initial body weight)]/feeding days; FR (feeding rate) = 100 × dry feed intake/[(final mean body weight + initial mean body weight)/2]/feeding days; FCR (feed conversion ratio) = total feed intake (dry matter)/(final biomass − initial biomass + biomass of dead fish); PER (protein efficiency ratio) = (final body weight − initial body weight)/total protein intake; SR (survival rate) = 100 × final fish number/initial fish number. CF (condition factor) = body weight [g]/(body length [cm])^3^; VSI (viscerosomatic index) = 100 × (visceral weight [g]/whole body weight [g]); HSI (hepatosomatic index) = 100 × (hepatopancreas weight [g]/whole body weight [g]); ISI (intestinosomatic index) = 100 × (intestinal weight [g]/whole body weight [g]).

**Table 5:** Effects of dietary fermented pineapple pomace inclusion level on hindgut morphology in largemouth bass.

	FP0	FP2	FP4	FP6	FP8	FP10	*p* Value
Villus height (μm)	790.89 ± 24.20 ^a^	796.84 ± 24.70 ^a^	920.70 ± 49.09 ^bc^	972.36 ± 47.64 ^c^	872.64 ± 36.60 ^abc^	818.47 ± 18.30 ^ab^	0.018
Villus width (μm)	135.13 ± 5.90 ^a^	157.73 ± 9.85 ^ab^	174.70 ± 5.58 ^b^	164.89 ± 4.74 ^b^	161.41 ± 2.33 ^b^	157.56 ± 10.22 ^ab^	0.035
Muscularis thickness (μm)	177.27 ± 9.72	173.92 ± 4.79	198.80 ± 8.04	189.52 ± 8.47	193.39 ± 6.35	187.12 ± 9.53	0.290

Values are expressed as the means ± standard error of the means (*n* = 3). Means within the same row with different superscripts indicate statistically significant differences among treatments (*p* < 0.05).

**Table 6:** Effects of dietary fermented pineapple pomace inclusion levels on the digestive and absorptive enzyme activities of largemouth bass.

	FP0	FP2	FP4	FP6	FP8	FP10	*p* Value
Pepsin (U/mg protein)	7.38 ± 0.50	7.76 ± 0.44	8.26 ± 0.09	7.30 ± 0.45	8.41 ± 0.49	7.41 ± 0.51	0.374
Trypsin (U/μg protein)	0.19 ± 0.01 ^a^	0.22 ± 0.01 ^ab^	0.28 ± 0.01 ^c^	0.23 ± 0.01 ^bc^	0.24 ± 0.01 ^bcd^	0.27 ± 0.02 ^cd^	0.002
Lipase (U/g protein)	38.32 ± 1.50 ^a^	55.19 ± 0.63 ^d^	53.21 ± 1.65 ^cd^	49.10 ± 1.16 ^bc^	45.78 ± 1.19 ^b^	51.06 ± 1.37 ^cd^	<0.001
Amylase (U/mg protein)	0.18 ± 0.01 ^a^	0.33 ± 0.05 ^d^	0.29 ± 0.02 ^bcd^	0.31 ± 0.03 ^cd^	0.21 ± 0.02 ^ab^	0.23 ± 0.02 ^abc^	0.009
Lactase (U/mg protein)	7.71 ± 0.54 ^a^	7.19 ± 0.18 ^a^	7.26 ± 0.27 ^a^	9.41 ± 0.71 ^bc^	8.22 ± 0.53 ^ab^	10.39 ± 0.33 ^c^	0.002
AKP (KU/g protein)	1.40 ± 0.02 ^a^	1.40 ± 0.02 ^a^	1.50 ± 0.07 ^ab^	1.67 ± 0.06 ^c^	1.51 ± 0.02 ^ab^	1.62 ± 0.02 ^bc^	0.003

Values are expressed as the means ± standard error of the means (*n* = 3). Means within the same row with different superscripts indicate statistically significant differences among treatments (*p* < 0.05).

**Table 7:** Effects of dietary fermented pineapple pomace inclusion levels on the intestinal mucosal permeability of largemouth bass.

	FP0	FP2	FP4	FP6	FP8	FP10	*p* Value
DAO (U/mL)	18.05 ± 0.48 ^c^	13.64 ± 0.57 ^a^	14.23 ± 0.24 ^a^	12.94 ± 0.83 ^a^	13.57 ± 0.13 ^a^	15.79 ± 0.09 ^b^	<0.001
D-LA (μmol/L)	36.57 ± 1.08 ^b^	30.91 ± 1.04 ^a^	33.70 ± 1.06 ^ab^	34.30 ± 1.03 ^b^	39.69 ± 0.60 ^c^	34.57 ± 0.6 ^b^	<0.001
ET-1 (pg/mL)	70.83 ± 1.21 ^b^	50.70 ± 1.42 ^a^	50.98 ± 0.69 ^a^	56.24 ± 2.00 ^a^	57.20 ± 2.58 ^a^	56.65 ± 2.29 ^a^	<0.001
LPS (ng/mL)	0.29 ± 0.01 ^c^	0.24 ± 0.01 ^a^	0.20 ± 0.02 ^a^	0.17 ± 0.01 ^ab^	0.23 ± 0.01 ^b^	0.23 ± 0.00 ^ab^	<0.001

Values are expressed as means ± standard error of the means (*n* = 3). Means within the same row with different superscripts indicate statistically significant differences among treatments (*p* < 0.05). DAO, diamine oxidase; D-LA, D-lactate. ET-1, endothelin-1; LPS, lipopolysaccharide.

**Table 8:** Effects of dietary fermented pineapple pomace inclusion levels on the intestinal chemical and immune barriers of largemouth bass.

	FP0	FP2	FP4	FP6	FP8	FP10	*p* Value
LZM (U/g protein)	27.68 ± 1.66 ^a^	28.86 ± 0.27 ^a^	34.95 ± 2.63 ^b^	38.88 ± 0.74 ^bc^	40.11 ± 0.53 ^c^	36.55 ± 0.72 ^bc^	<0.001
MUC2 (ng/g protein)	40.06 ± 0.93 ^a^	44.90 ± 0.74 ^ab^	48.04 ± 0.63 ^bc^	52.11 ± 2.08 ^c^	46.53 ± 2.37 ^b^	51.81 ± 1.80 ^c^	0.001
IgM (mg/g protein)	0.18 ± 0.01 ^a^	0.20 ± 0.01 ^a^	0.21 ± 0.01 ^a^	0.20 ± 0.00 ^a^	0.25 ± 0.02 ^b^	0.22 ± 0.01 ^ab^	0.032
sIgT (mg/g protein)	1.48 ± 0.06 ^a^	1.37 ± 0.03 ^a^	1.72 ± 0.05 ^b^	2.11 ± 0.06 ^c^	1.78 ± 0.06 ^b^	1.79 ± 0.08 ^b^	<0.001
IL-1β (ng/g protein)	6.91 ± 0.06 ^b^	7.16 ± 0.25 ^b^	7.22 ± 0.32 ^b^	5.87 ± 0.55 ^a^	5.54 ± 0.20 ^a^	5.06 ± 0.16 ^a^	<0.001
IL-8 (ng/g protein)	7.08 ± 0.37 ^b^	5.11 ± 0.42 ^a^	5.96 ± 0.40 ^ab^	6.12 ± 0.41 ^ab^	5.86 ± 0.18 ^b^	6.08 ± 0.21 ^ab^	0.040
TNF-α (ng/g protein)	64.01 ± 2.18 ^c^	49.53 ± 0.78 ^b^	26.08 ± 1.98 ^a^	46.93 ± 3.24 ^b^	42.85 ± 3.00 ^b^	63.32 ± 1.94 ^c^	<0.001
IL-10 (ng/g protein)	43.56 ± 0.52 ^b^	39.40 ± 0.96 ^a^	48.35 ± 1.19 ^c^	52.67 ± 2.05 ^d^	46.72 ± 0.82 ^bc^	53.67 ± 0.42 ^d^	<0.001
TGF-β (μg/g protein)	0.30 ± 0.02	0.32 ± 0.01	0.29 ± 0.01	0.33 ± 0.02	0.33 ± 0.02	0.36 ± 0.01	0.062

Values are expressed as means ± standard error of the means (*n* = 3). Means within the same row with different superscripts indicate statistically significant differences among treatments (*p* < 0.05). LZM, lysozyme; MUC2, mucin 2; IgM, immunoglobulin M; sIgT, secretory immunoglobulin T; IL-1β, interleukin-1 beta; IL-8, interleukin-8; TNF-α, tumor necrosis factor-alpha; IL-10, interleukin-10; TGF-β, transforming growth factor-beta.

**Table 9:** Effects of dietary fermented pineapple pomace inclusion levels on the antioxidant parameters in largemouth bass.

	FP0	FP2	FP4	FP6	FP8	FP10	*p* Value
Serum							
SOD (U/mg protein)	10.92 ± 0.91 ^a^	13.86 ± 1.66 ^b^	12.02 ± 0.78 ^b^	9.39 ± 0.85 ^a^	10.36 ± 0.95 ^a^	9.75 ± 0.36 ^a^	<0.001
CAT (U/mg protein)	12.80 ± 0.49 ^a^	23.96 ± 0.81 ^d^	23.78 ± 0.28 ^d^	18.08 ± 0.63 ^b^	20.78 ± 0.73 ^c^	20.44 ± 0.36 ^c^	<0.001
GSH (U/g protein)	1.17 ± 0.05 ^b^	0.76 ± 0.03 ^a^	1.26 ± 0.10 ^b^	1.27 ± 0.07 ^b^	1.31 ± 0.04 ^b^	1.26 ± 0.05 ^b^	<0.001
T-AOC (mmol/g protein)	0.37 ± 0.03 ^a^	0.48 ± 0.02 ^ab^	0.61 ± 0.04 ^b^	0.59 ± 0.05 ^b^	0.56 ± 0.07 ^b^	0.47 ± 0.05 ^ab^	0.025
MDA (nmol/mg protein)	37.04 ± 1.27 ^c^	16.63 ± 0.93 ^a^	26.74 ± 1.32 ^b^	33.04 ± 0.93 ^c^	37.18 ± 1.63 ^c^	32.91 ± 1.66 ^c^	<0.001
Intestine							
SOD (U/mg protein)	11.83 ± 0.68	12.13 ± 0.74	11.24 ± 0.61	13.48 ± 1.18	12.74 ± 0.91	12.64 ± 0.64	0.504
CAT (U/mg protein)	0.66 ± 0.06 ^a^	0.76 ± 0.12 ^ab^	1.55 ± 0.13 ^e^	1.38 ± 0.15 ^de^	1.15 ± 0.09 ^cd^	1.02 ± 0.08 ^bc^	<0.001
POD (U/g protein)	17.84 ± 0.45 ^a^	19.91 ± 0.47 ^ab^	21.39 ± 1.16 ^b^	37.72 ± 0.98 ^d^	27.00 ± 0.95 ^c^	27.54 ± 0.90 ^c^	<0.001
T-AOC (mmol/g protein)	47.75 ± 1.70 ^a^	49.78 ± 1.33 ^a^	50.58 ± 1.18 ^a^	59.29 ± 1.69 ^bc^	56.44 ± 1.87 ^b^	62.45 ± 0.79 ^c^	<0.001
MDA (nmol/mg protein)	10.33 ± 0.67 ^c^	9.24 ± 0.47 ^bc^	5.69 ± 0.36 ^a^	5.05 ± 0.41 ^a^	6.10 ± 0.46 ^a^	8.39 ± 0.18 ^b^	<0.001
Liver							
SOD (U/mg protein)	31.12 ± 1.35	35.72 ± 0.85	33.69 ± 1.73	37.26 ± 2.71	37.60 ± 1.95	30.68 ± 1.66	0.068
CAT (U/mg protein)	7.05 ± 0.51 ^ab^	5.95 ± 0.29 ^a^	6.95 ± 0.59 ^ab^	7.84 ± 0.53 ^bc^	8.07 ± 0.70 ^bc^	8.92 ± 0.63 ^c^	0.037
POD (U/g protein)	21.35 ± 0.54 ^b^	16.42 ± 0.40 ^a^	22.20 ± 1.42 ^b^	23.93 ± 1.12 ^b^	21.67 ± 1.48 ^b^	23.97 ± 1.40 ^b^	0.006
T-AOC (mmol/g protein)	42.25 ± 0.67 ^a^	52.30 ± 3.69 ^bc^	53.45 ± 1.22 ^bc^	57.74 ± 3.50 ^c^	55.64 ± 1.75 ^bc^	47.88 ± 3.51 ^ab^	0.016
MDA (nmol/mg protein)	0.98 ± 0.04 ^b^	0.72 ± 0.03 ^a^	0.80 ± 0.09 ^a^	0.88 ± 0.02 ^a^	1.16 ± 0.15 ^b^	1.41 ± 0.07 ^c^	<0.001

Values are expressed as the means ± standard error of the means (*n* = 3). Means within the same row with different superscripts indicate statistically significant differences among treatments (*p* < 0.05).

## Data Availability

The data supporting the findings of this study are available from the corresponding author upon reasonable request.

## Abbreviations

The following abbreviations are used in this manuscript:

FPP	Fermented Pineapple Pomace
PP	Pineapple Pomace
TEM	Transmission Electron Microscopy
ET-1	Endothelin-1
D-LA	D-Lactate
LPS	Lipopolysaccharide
DAO	Diamine Oxidase
LZM	Lysozyme
MUC2	Mucin 2
IgM	Immunoglobulin M
sIgT	Secretory Immunoglobulin T
IL-1β	Interleukin-1 Beta
IL-8	Interleukin-8
TNF-α	Tumor Necrosis Factor-Alpha
IL-10	Interleukin-10
TGF-β	Transforming Growth Factor-Beta
SOD	Superoxide Dismutase
CAT	Catalase
GSH	Reduced Glutathione
T-AOC	Total Antioxidant Capacity
MDA	Malondialdehyde
POD	Peroxidase
IBW	Initial Body Weight
FBW	Final Body Weight
WGR	Weight Gain Rate
SGR	Specific Growth Rate
FR	Feeding Rate
FCR	Feed Conversion Ratio
PER	Protein Efficiency Ratio
SR	Survival Rate
CF	Condition Factor
VSI	Viscerosomatic Index
HSI	Hepatosomatic Index
ISI	Intestosomatic Index
ILI	Intestinal Length Index
*β-actin*	Beta-Actin
*ZO-1*	Zonula Occludens Protein 1
TJ	Tight Junctions
MV	Microvilli
